# Supplementary material for: Major data analysis errors invalidate cancer microbiome findings
Source: bioRxiv. 2023 Jul 31:2023.07.28.550993. Preprint. [Version 1] doi: 10.1101/2023.07.28.550993 (PMC10418105; doi:10.1101/2023.07.28.550993)
Supplement: Supplement 1 [file media-1.pdf]

Supplementary Tables and Figures

Table S1. Average number of reads in the top 20 most abundant genera for TCGA-BLCA as computed in the Poore *et al*. study, averaged over 129 WGS primary tumor and 27 solid tissue normal samples.

**Genus**	**Average read count per sample, Poore *et al.***	**Average read count per sample, this study**
Streptococcus	560212	36
Mycobacterium	410968	6.1
Staphylococcus	240880	282
Waddlia	55280	0
Bacillus	53659	4
Escherichia	50207	3.4
Bordetella	46075	0.4
Pseudomonas	44186	133
Pseudoalteromonas	40837	0.2
Vibrio	34216	0.2
Streptomyces	28317	2.9
Piscirickettsia	24818	0
Klebsiella	21069	2.1
Microbacterium	16773	0.9
Xanthomonas	15681	22
Shigella	14208	0.7
Acinetobacter	12562	8.6
Bacteroides	12013	7.7
Neisseria	11988	0.4
Salmonella	11234	0.3

Table S2. Average number of reads in the top 20 most abundant genera for TCGA-BLCA as measured in this study, averaged over 129 WGS primary tumor and 27 solid tissue normal samples.

**Genus**	**Average read count per sample, this study**	**Average read count per sample, Poore *et al.***
Enterococcus	447	9899
Veillonella	385	726
Staphylococcus	282	240880
Aerococcus	165	1756
Pseudomonas	133	44186
Peptoniphilus	95	737
Finegoldia	80	258
Stenotrophomonas	64	846
Anaerococcus	50	2451
Prevotella	49	1062
Streptococcus	36	560212
Cupriavidus	36	137

Actinotignum	31	153
Citrobacter	25	695
Cutibacterium	24	0
Betapolyomavirus	22	0
Xanthomonas	22	15681
Erysipelatoclostridium	22	1.1
Campylobacter	18	2185
Eimeria	12	0

Table S3. Average read counts for the top 20 genera, ranked by weights assigned by the machine learning classifier using the "all putative contaminants removed" dataset and classifying BLCA primary tumor samples versus all other tumor types. Counts are averaged over 129 WGS primary tumor and 27 solid tissue normal samples.

**Genus**	**Average read count, Poore *et al.***	**Average read count, this study**
Nitrospira	7.5	0
Elizabethkingia	460	0.1
Leptospira	3053	0
Campylobacter	2185	18
Histophilus	162	0
Capnocytophaga	56	0.2
Chelativorans	0	0
Sediminibacterium	2.6	0
Scardovia	0.6	0
Lysobacter	47	0.2
Stomatobaculum	0	0
Gallibacterium	20	0
Turicella	0.1	0
Betaretrovirus	0.2	0
Exiguobacterium	789	0
Wolbachia	291	0
Bacteroides	12013	7.7
Alphapapillomavirus	403	0.1
Hydrogenibacillus	17	0
Candidatus Stoquefichus	75	0

Table S4. Average read counts for the top 20 genera, ranked by weights assigned by the machine learning classifier using the "all putative contaminants removed" dataset and classifying BLCA primary tumor samples versus solid tissue normal samples. Counts are averaged over 129 WGS primary tumor and 27 solid tissue normal samples.

**Genus**	**Average read count, Poore *et al.***	**Average read count, this study**

Lachnoclostridium	18	0.2
Methylobacter	1.7	0
Marinitoga	157	0
Vibrio	34216	0.2
Crocinitomix	1.3	0
Flammeovirga	2.3	0
Desulfobacter	0.4	0
Paeniclostridium	148	0.1
Aliihoeflea	0	0
Cellulomonas	73	0.1
Candidatus Nitrosopelagicus	0.2	0
Tymovirus	10	0
Betapartitivirus	72	0
Microvirga	77	0
Salsuginibacillus	0.6	0
Nepovirus	5.7	0
Aeromonas	435	1.9
Klebsiella	21069	2.1
Pusillimonas	0.5	0
Candidatus Evansia	1.7	0

Table S5. Average number of reads in the top 20 most abundant genera for TCGA-HNSC as computed in the Poore *et al.* study, averaged over 334 WGS samples.

**Genus**	**Average read count, Poore *et al.***	**Average read count, this study**
Streptococcus	1335308	2041
Mycobacterium	1001984	22.9
Staphylococcus	670494	121
Pseudomonas	273551	5685
Escherichia	212531	64.4
Mesorhizobium	158289	115
Waddlia	144680	0.0
Bacillus	137082	11.6
Neisseria	128526	1651
Pseudoalteromonas	116176	1.0
Streptomyces	96294	16.8
Vibrio	93027	2.8
Bordetella	84532	5.3
Shigella	70859	1.6
Piscirickettsia	66821	0.0
Treponema	63757	4774
Fusobacterium	57873	10003

Salmonella	53463	1.2
Klebsiella	51586	34.5
Microbacterium	50001	10.1

Table S6. Average number of reads in the top 20 most abundant genera for TCGA-HNSC as measured in this study, averaged over 334 WGS samples.

**Genus**	**Average read count, this study**	**Average read count, Poore *et al.***
Fusobacterium	10003	57873
Capnocytophaga	5981	13925
Prevotella	5706	47835
Pseudomonas	5685	273551
Treponema	4774	63757
Campylobacter	2102	26697
Streptococcus	2041	1335308
Neisseria	1651	128526
Veillonella	1328	3826
Haemophilus	1270	6870
Sphingomonas	1094	12979
Leptotrichia	721	1893
Stenotrophomonas	590	2646
Parvimonas	546	4062
Tannerella	428	2887
Porphyromonas	410	17431
Selenomonas	349	1111
Rothia	331	1027
Bifidobacterium	323	1522
Bradyrhizobium	301	2852

Table S7. Average read counts for the top 20 genera, ranked by weights assigned by the machine learning classifier using the "all putative contaminants removed" dataset and classifying HNSC primary tumor samples versus all other tumor types. Counts are averaged over 170 WGS primary tumor, 140 blood derived normal, and 24 solid tissue normal samples.

**Genus**	**Average read count, Poore *et al.***	**Average read count, this study**

Microvirga	209	1.8
Gemmata	13385	0.4
Desulfomicrobium	1.7	0.8
Exiguobacterium	1331	0.3
Nitrosopelagicus	0.4	0.0
Alphapapillomavirus	8577	0.4
Phenylobacterium	21	4.0
Klebsiella	51586	35
Plantibacter	0.3	0.3
Terracoccus	5.0	0.0
Marichromatium	0.8	0.3
Betapartitivirus	159	0.0
Gottschalkia	2.2	0.4
Acidithiobacillus	142	0.4
Desulfococcus	24868	0.2
Epilithonimonas	5.8	0.0
Luteibacter	1036	0.5
Spiroplasma	356	0.7
Mannheimia	429	2.2
Roseivirga	4.2	0.1

Tables S8, S9, and S10 (separate files): These tables contains all read counts, reported at the genus level, from the bladder cancer (BLCA), head and neck cancer (HNSC), and breast cancer (BRCA) samples from TCGA. The tables include read counts for bacteria, archaea, and viruses. All samples were classified against a KrakenUniq database as described in Methods. **Table S8** (156 x 1063) has read counts for 156 BLCA samples, including 129 primary tumor and 27 solid tissue normal samples, filtered to remove human reads as described in Methods.
Overall, 1,063 genera were identified (i.e., contained at least one non-zero count) in the 156 samples. **Table S9** (334 x 1573) contains read counts for 334 HNSC WGS, including 170 primary tumor samples, 140 blood derived normal samples, and 24 solid tissue normal samples, filtered to remove human reads as described in Methods. Overall, 1,573 genera were identified (i.e., contained at least one non-zero count) across the 334 samples. **Table S10** (238 x 1,200) contains read counts for 238 BRCA WGS samples, including 114 primary tumor samples, 106 blood derived normal samples, 16 solid tissue normal samples, and 2 metastatic samples, filtered to removed human reads as described in Methods. Overall, 1,200 genera were identified (i.e., contained at least one non-zero count) across the 238 samples.

Supplementary Data File 1. List of all species contained in the Kraken database used in this study.
Supplementary Data File 2. List of all genera contained in the Kraken database used in this study.

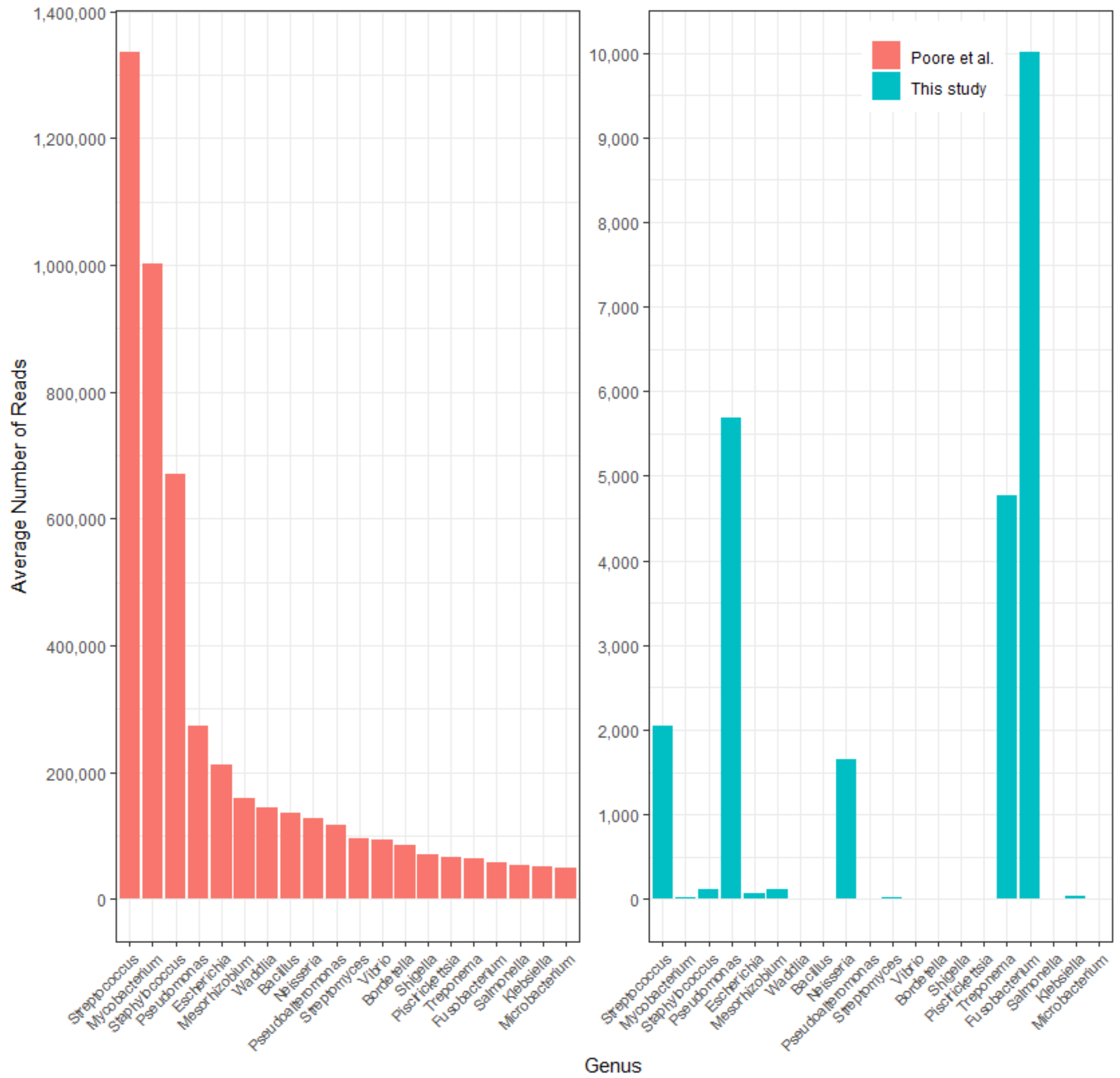

Figure S1. Average number of reads per sample in head and neck cancer (HNSC) in the top 20 most-abundant genera reported in Poore *et al*. (left), averaged over 170 WGS primary tumor, 140 blood derived normal, and 24 solid tissue normal samples. On the right are the counts for the same genera, in the same order, as computed in our re-analysis. Note that the y-axis scales are different by a factor of ~150. The x-axis shows genus names.

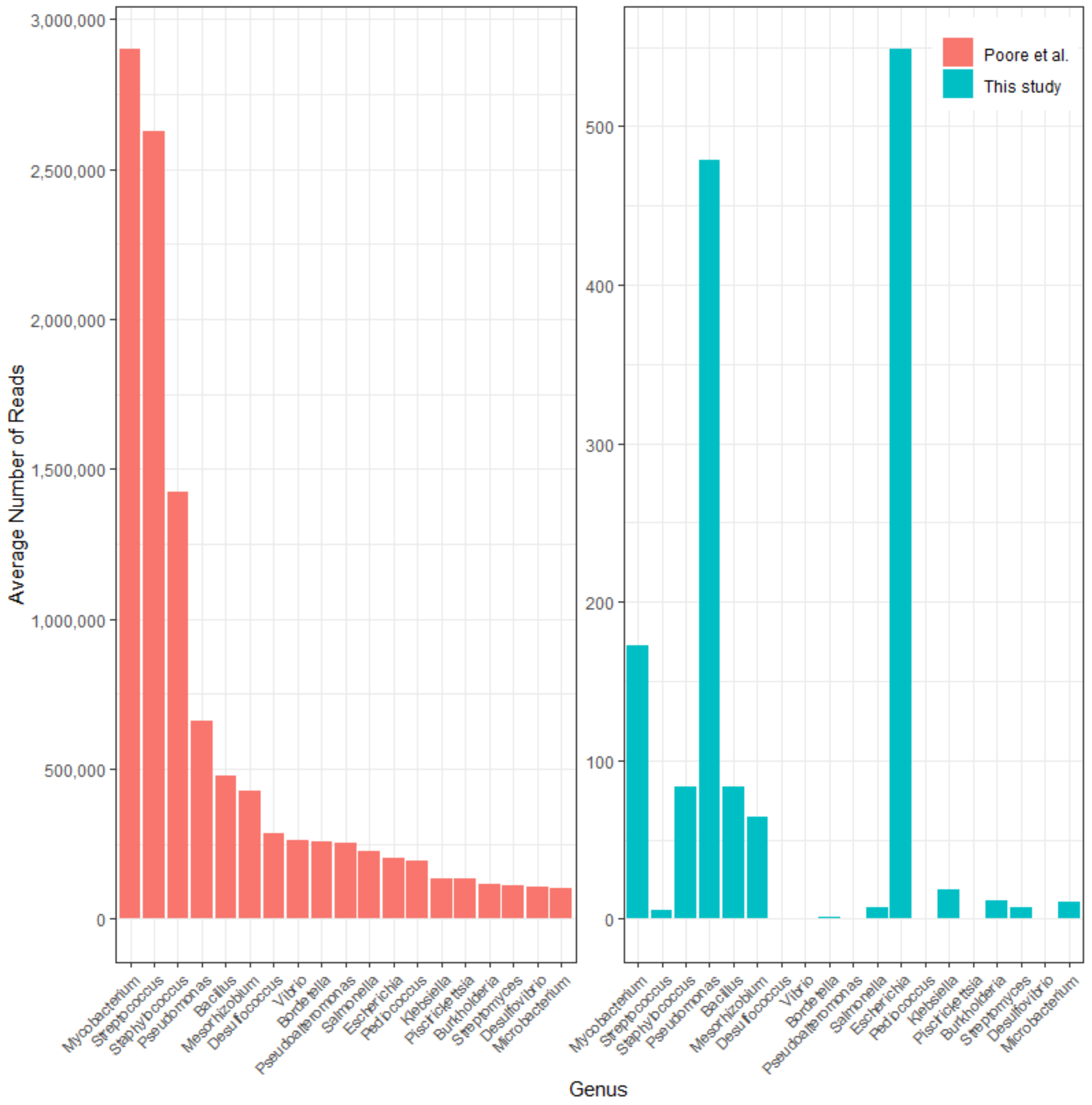

Figure S2. Average number of reads per sample in breast invasive carcinoma (BRCA) in the top 20 most-abundant genera reported in Poore *et al*. (left), averaged over 238 breast cancer WGS samples. On the right are the counts for the same genera, in the same order, as computed in our re-analysis. Note that the y-axis scales are different by a factor of ~5000. The x-axis shows genus names.
